# HIV-related outcome disparities between transgender women living with HIV and cisgender people living with HIV served by the Health Resources and Services Administration’s Ryan White HIV/AIDS Program: A retrospective study

**DOI:** 10.1371/journal.pmed.1003125

**Published:** 2020-05-28

**Authors:** Pamela W. Klein, Demetrios Psihopaidas, Jessica Xavier, Stacy M. Cohen

**Affiliations:** 1 Health Resources and Services Administration, HIV/AIDS Bureau, Rockville, Maryland, United States of America; 2 Independent Consultant, Silver Spring, Maryland, United States of America; University of California, San Francisco, UNITED STATES

## Abstract

**Background:**

In the United States, approximately one-fifth of transgender women are living with HIV—nearly one-half of Black/African American (Black) transgender women are living with HIV. Limited data are available on HIV-related clinical indicators among transgender women. This is because of a lack of robust transgender data collection and research, especially within demographic subgroups. The objective of this study was to examine retention in care and viral suppression among transgender women accessing the Health Resources and Services Administration’s (HRSA) Ryan White HIV/AIDS Program (RWHAP)-supported HIV care, compared with cisgender women and cisgender men.

**Methods and findings:**

We assessed the association between gender (cisgender or transgender) and (1) retention in care and (2) viral suppression using 2016 client-level RWHAP Services Report data. Multivariable modified Poisson regression models adjusting for confounding by age, race, health care coverage, housing, and poverty level, overall and stratified by race/ethnicity, were used to calculate adjusted prevalence ratios (aPRs) and 95% confidence intervals (CIs). In 2016, the RWHAP served 6,534 transgender women (79.8% retained in care, 79.0% virally suppressed), 143,173 cisgender women (83.7% retained in care, 84.0% virally suppressed), and 382,591 cisgender men (81.0% retained in care, 85.9% virally suppressed). Black transgender women were less likely to be retained in care than Black cisgender women (aPR: 0.95, 95% CI: 0.92–0.97, *p <* 0.001). Black transgender women were also less likely to reach viral suppression than Black cisgender women (aPR: 0.55, 95%I CI: 0.41–0.73, *p* < 0.001) and Black cisgender men (aPR: 0.55, 95% CI: 0.42–0.73, *p <* 0.001). A limitation of the study is that RWHAP data are collected for administrative, not research, purposes, and clinical outcome measures, including retention and viral suppression, are only reported to the RWHAP for the approximately 60% of RWHAP clients engaged in RWHAP-supported outpatient medical care.

**Conclusions:**

In this study, we observed disparities in HIV clinical outcomes among Black transgender women. These results fill an important gap in national HIV data about transgender people with HIV. Reducing barriers to HIV medical care for transgender women is critical to decrease disparities among this population.

## Abstract 2

In an observational study of data from a 2016 report, Klein and colleagues investigate associations between racial and ethnic disparities and gender identity with HIV clinical outcomes among individuals in the US served by HRSA's Ryan White HIV/AIDS Program

## Author summary

**Why was this study done?:**

- Transgender people often have difficulty accessing HIV care, which can lead to poor health outcomes and increased HIV transmission.
- Large-scale national surveillance data have not adequately assessed HIV outcomes among transgender people.
- The Health Resources and Services Administration (HRSA)’s Ryan White HIV/AIDS Program (RWHAP) serves more than 11,000 transgender people, which is more people than were included in previous studies.
- This study was conducted to compare HIV clinical outcomes among transgender and cisgender people with HIV accessing HIV medical care through the RWHAP.

**What did the researchers do and find?:**

- We analyzed patient data from the RWHAP to determine how many clients were retained in care and virally suppressed in 2016 by gender and race/ethnicity.
- Transgender women who received HIV medical care services through the RWHAP were younger, more likely to identify as a racial/ethnic minority, and less likely to have stable housing than cisgender men and cisgender women.
- Black transgender women were significantly less likely to reach viral suppression than both Black cisgender men and Black cisgender women.

**What do these findings mean?:**

- In this study, Black transgender women were less likely to reach optimal HIV health outcomes than cisgender people.
- The RWHAP provides HIV care and support services to a large number of transgender people, filling an important data and programmatic gap.
- Our results suggest that initiatives such as those supported by the RWHAP to reduce health disparities between transgender and cisgender people may be beneficial to the health of transgender people with HIV and prevent HIV transmission.

## Introduction

Transgender people experience a disproportionately heavy burden of HIV and continue to be an underserved community. Transgender women, in particular, are nearly 50 times as likely to have HIV as other people of reproductive age [1]. In the United States, approximately one-fifth of transgender women are living with HIV—nearly one-half of Black/African American (Black) transgender women are living with HIV [2,3]. Ongoing HIV care and treatment are vital to reducing morbidity and mortality for people living with HIV (PLWH) [4,5]. However, compared with their cisgender (non-transgender) counterparts, transgender women and men are less likely to be aware of their HIV status, are more likely to delay HIV testing, and are disproportionately more likely to experience barriers to accessing HIV-specific healthcare after HIV diagnosis [2,6,7]. Intersecting social and structural challenges can serve as barriers to HIV care for transgender PLWH, including individual-level factors such as incarceration, homelessness, intimate partner violence, and a fear of disclosing transgender identity, and systems-level factors such as discrimination by healthcare providers and clinical staff, healthcare providers’ lack of culturally relevant healthcare practices for transgender clients, and a lack of sufficient health care coverage [8–13].

In addition to social and structural challenges, transgender PLWH also face barriers in obtaining and maintaining access to medical care. These barriers to medical care access correlate with HIV health outcomes. Although no national data on HIV outcomes among transgender people are available, observational studies indicate that transgender PLWH are less likely to be retained in primary medical care, to receive and adhere to antiretroviral treatment (ART), and to reach viral suppression compared to cisgender PLWH [14–22]. Evidence suggests that racial/ethnic minority transgender women, specifically Hispanic/Latina and Black transgender women, face additional barriers to healthcare access including HIV-specific healthcare [23]. Limited data are available on HIV-related clinical indicators among transgender women because of a lack of robust transgender data collection and research, especially within demographic subgroups [24]. Furthermore, many studies are limited by small sample sizes and inconsistent comparison groups, resulting in insufficient power for broad conclusions and difficulty in synthesizing results across studies.

Given the lack of national HIV surveillance data on HIV clinical outcomes among transgender women, the Health Resources and Services Administration’s HIV/AIDS Bureau (HRSA HAB) Ryan White HIV/AIDS Program (RWHAP) presents a unique opportunity to examine HIV clinical outcomes (i.e., retention in care and viral suppression) among a large sample of transgender women in the United States (U.S.). The RWHAP is a comprehensive system of HIV care, treatment, and support services that addresses the needs of low-income PLWH in the US and provides sufficient power to identify differences in HIV clinical outcomes within racial/ethnic subgroups. Client-level data from the RWHAP are reported through the RWHAP Services Report (RSR)—one of the largest client-level data sources for HIV care and treatment in the US. The RSR collects comprehensive gender information including 3 subcategories for transgender: transgender male-to-female (MTF), transgender female-to-male (FTM), and transgender unknown (which may include transgender people who do not identify as MTF or FTM) [25]. The RWHAP considers the term transgender an umbrella term used to identify people whose sex assigned at birth does not match their current gender identity or expression.

In this analysis, we examine differences in 2 HIV-related clinical indicators (retention in care and viral suppression) among transgender women (MTF) accessing RWHAP-supported HIV outpatient ambulatory health services (OAHS), compared separately with cisgender women and cisgender men. We then investigate whether racial/ethnic disparities exist in the association between gender identity and HIV clinical outcomes. Identifying disparities in HIV-related health outcomes can help support the development and implementation of interventions designed for specific demographic populations at greatest risk for poor health outcomes.

## Methods

### Data source and study population

Data for this analysis are from the 2016 RSR. The RSR data set is HRSA HAB’s primary source of annual, client-level RWHAP data used to assess the numbers and demographics of clients receiving services as well as their HIV-related outcomes. Each year, grant recipients and subrecipients that receive RWHAP funds to provide core medical or support services are required to submit data to HRSA HAB. Client-level RSR data are reported by more than 2,000 grant recipients and subrecipients in the US, including the 50 states, the District of Columbia, and 3 territories (Guam, Puerto Rico, and the US Virgin Islands) [25]. This analysis includes RSR data for all adult and adolescent clients (i.e., aged ≥13 years) living with HIV served by the RWHAP Parts A, B, C, and D during the 2016 calendar year.

Data collection through RSR and other RWHAP data sources is a routine program activity, and the data are used for program monitoring, improvement, evaluation, and policy purposes only. Therefore, it is not human subject research and does not require institutional review board (IRB) review and approval. This study is reported as per the Strengthening the Reporting of Observational Studies in Epidemiology (STROBE) guideline (S1 STROBE Checklist). This study was prospectively conceived and planned in January 2018, with analyses conducted through October 2018. Based on reviewer comments, we revised the statistical modeling approach, the results of which are presented in this manuscript.

### Definitions

Data from the RSR use a two-step method for determining gender identity, which first identifies sex assigned at birth, followed by current gender identity (male, female, FTM, MTF, and transgender unknown). Gender-related data are based on the client’s self-reported gender identification. For this analysis, MTF transgender clients (transgender women) were compared separately to cisgender women and cisgender men. Transgender men were excluded from this analysis because of small numbers; although 430 transgender men were served by the RWHAP in 2016, fewer than 300 transgender men met the denominator criteria for measuring retention in care and viral suppression.

The 2 outcomes in this analysis were (1) annual retention in HIV medical care and (2) viral suppression. Retention in HIV medical care was assessed among PLWH with at least 1 OAHS visit during the calendar year; clients were retained in care if they had at least 2 OAHS visit dates that were at least 90 days apart in 2016, with the first visit occurring before September 1. Viral suppression was assessed among PLWH with at least 1 OAHS visit during the calendar year and at least 1 viral load test; clients were virally suppressed if their most recent reported HIV RNA test result was <200 copies/mL. These outcomes are consistent with HIV/AIDS Bureau core performance measures, which are used by RWHAP grant recipients to assess quality of care and drive quality improvement initiatives [26,27].

Client demographics (e.g., age, poverty level) were considered potential confounders for the association between gender and HIV clinical outcomes. For this analysis, client age and the number of OAHS visits were measured as continuous variables. Race and ethnicity information was according to Office of Management and Budget (OMB) reporting standards categorized as: Black (non-Hispanic/Latino), Hispanic/Latino, White (non-Hispanic/Latino), and Other (non-Hispanic/Latino; i.e., American Indian/Alaskan Native, Asian, Native Hawaiian/Pacific Islander, or multiple races) [28]. RWHAP grant recipients are expected to make every effort to obtain and report race and ethnicity based on each client’s self-report. Poverty level characterizes the client’s household income as a percentage of the federal poverty level (FPL) and was reported as a 5-level variable: 0% to 100%, 101% to 138%, 139% to 250%, 251% to 400%, and >400% FPL. RWHAP grant recipients and subrecipients report all sources of health care coverage that each client had for any part of the calendar year. For descriptive analysis, health care coverage was categorized into 10 subgroups: private insurance purchased by an employer (private employer), private insurance purchased by an individual (private individual), Medicare, Medicaid, Medicare and Medicaid (dual eligibility), Veterans Administration, Indian Health Service, other plan, multiple coverages, and no coverage. For regression analysis, this categorization was collapsed to a 4-level variable: no health care coverage, public coverage (Medicare, Medicaid, Medicare and Medicaid, Veterans Administration, Indian Health Service), private coverage (employer or individual), and other (other plan, multiple coverages). If a client lacked health care coverage for part of the year but also had some type of health care coverage, the client would be classified by the type of health care coverage. If a client had multiple forms of health care coverage in a single calendar year, with the exception of coverage by both Medicare and Medicaid, the client would be classified as “multiple coverages.” Housing status was measured at the last client interaction of the calendar year and categorized as stable, temporary, or unstable. Region was defined by the US Census Bureau based on the state in which a client received RWHAP services [29]. Clients receiving services in multiple states were coded as “missing”; these clients make up less than 2% of the total RWHAP population. Minimal data were missing on race/ethnicity (approximately 1%), poverty level (approximately 3.5%), health care coverage (approximately 2.5%), and housing status (approximately 3.5%); the amount of missingness for each variable was comparable for transgender women, cisgender women, and cisgender men; clients with missing data were excluded from regression analyses.

### Statistical analysis

Differences in sociodemographic characteristics between transgender women, cisgender women, and cisgender men were examined for the entire RWHAP client population aged ≥13 years and for the subset of RWHAP clients aged ≥13 years with at least 1 OAHS visit.

The associations between gender, retention in care, and viral suppression were assessed descriptively with a Mantel–Hansel chi-square test (results in Table 3) and with multivariable modified Poisson regression with a robust variance estimator (results in Table 4). Based on literature supporting significantly different lived experiences impacting healthcare access and HIV care outcomes by race/ethnicity, race/ethnicity was considered an a priori effect measure modifier and all results were stratified by race/ethnicity (White, Black, and Hispanic/Latino) [11,30–35]. Age, poverty level, health care coverage, and housing status were assessed as potential confounders of the total direct effect using directed acyclic graphs and an assessment of multicollinearity. After this assessment, all 4 variables were included in the final models. The final models resulted in adjusted prevalence ratios and associated 95% confidence intervals (CIs). All statistical analyses were completed with SAS 9.3 (https://www.sas.com/).

## Results

### RWHAP client demographics

In 2016, the RWHAP served 6,534 transgender women, 143,173 cisgender women, and 382,591 cisgender men (Table 1). Transgender women were younger (median age of 38 years, interquartile range [IQR]: 30–48 years) than cisgender women (median age 48 years, IQR: 39–56) and cisgender men (median age 48 years, IQR: 36–55). Racial/ethnic minority populations accounted for 88.7% of transgender women, 83.8% of cisgender women, and 68.8% of cisgender men. Transgender and cisgender women had lower incomes than cisgender men, with 77.9% of transgender women and 72.0% of cisgender women living at or below the 100% FPL, compared with 58.9% of cisgender men. Among transgender women, 48.3% had Medicaid coverage and 22.1% had no health care coverage; 42.1% of cisgender women had Medicaid and 16.7% had no health care coverage; and 28.3% of cisgender men had Medicaid and 21.9% had no health care coverage. A higher percentage of transgender women had temporary (14.2%) or unstable (11.3%) housing in 2016, compared with cisgender women (7.7% and 4.2%, respectively) and cisgender men (9.1% and 5.4%, respectively). A lower percentage of transgender women received RWHAP services in the south (32.6%) than cisgender women (47.6%) and cisgender men (42.0%).

**Table 1:** Transgender women, cisgender women, and cisgender men served by the HRSA RWHAP, 2016.

Sociodemographic characteristics	Transgender women (*N* = 6,534)	Cisgender women (*N* = 143,173)	Cisgender men (*N* = 382,591)
**Age (years), median (IQR)**	38 (30–48)	48 (39–56)	48 (36–55)
**Race/ethnicity**^**a**^**, *n* (%)**			
Black	3,513 (54.4)	87,615 (61.5)	156,465 (41.1)
Hispanic/Latino	1,896 (29.3)	28,072 (19.7)	92,921 (24.4)
White	732 (11.3)	23,109 (16.2)	118,559 (31.2)
Other	322 (5.0)	3,678 (2.6)	12,364 (3.3)
**Poverty level, *n* (%)**			
0%–100%	4,910 (77.9)	99,446 (72.0)	217,094 (58.9)
101%–138%	611 (9.7)	14,762 (10.7)	46,438 (12.6)
139%–250%	551 (8.7)	17,061 (12.4)	66,834 (18.1)
251%–400%	164 (2.6)	5,473 (4.0)	28,172 (7.6)
>400%	64 (1.0)	1,358 (1.0)	10,208 (2.8)
**Health care coverage, *n* (%)**			
Private employer	161 (2.5)	9,535 (6.8)	35,681 (9.5)
Private individual	318 (5.0)	7,827 (5.6)	32,717 (8.8)
Medicare	361 (5.7)	13,025 (9.3)	41,977 (11.2)
Medicaid	3,057 (48.3)	58,940 (42.1)	105,981 (28.3)
Medicare and Medicaid	380 (6.0)	11,757 (8.4)	27,746 (7.4)
Veterans Administration	5 (0.1)	183 (0.1)	1,282 (0.3)
Indian Health Service	1 (0.0)	63 (0.1)	114 (0.0)
Other plan	132 (2.1)	2,306 (1.7)	7,233 (1.9)
No coverage	1,395 (22.1)	23,414 (16.7)	81,870 (21.9)
Multiple coverages	517 (8.2)	12,838 (9.2)	39,478 (10.6)
**Housing status, *n* (%)**			
Stable	4,700 (74.6)	121,998 (88.2)	315,702 (85.4)
Temporary	893 (14.2)	10,618 (7.7)	33,730 (9.1)
Unstable	709 (11.3)	5,773 (4.2)	20,085 (5.4)
**Region, *n* (%)**			
Northeast	2,069 (32.2)	42,245 (30.8)	89,058 (24.1)
Midwest	871 (13.5)	15,828 (11.5)	52,435 (14.2)
South	2,098 (32.6)	65,365 (47.6)	155,163 (42.0)
West	1,395 (21.7)	13,929 (10.1)	72,613 (19.7)

^a^Hispanics/Latinos can be of any race

Transgender women missing data on race/ethnicity (*n* = 71), poverty level (*n* = 234), health care coverage (*n* = 207), housing status (*n* = 232), and region (*n* = 100)

Cisgender women missing data on race/ethnicity (*n* = 699), poverty level (*n* = 5,073), health care coverage (*n* = 3,285), housing status (*n* = 4,784), and region (*n* = 5,806)

Cisgender men missing data on race/ethnicity (*n* = 2,282), poverty level (*n* = 13,845), health care coverage (*n* = 8,512), and housing status (*n* = 13,074), and region (*n* = 13,322)

**Abbreviations:** HRSA RWHAP, Heath Resources and Services Administration’s Ryan White HIV/AIDS Program; IQR, interquartile range

The proportion of all RWHAP clients who accessed OAHS and the median number of OAHS visits per year was similar across groups, with 64.2% of transgender women, 68.8% of cisgender women, and 61.6% of cisgender men accessing at least 1 OAHS visit in 2016 (Table 2). The percentage distributions across subpopulations of transgender women, cisgender women, and cisgender men who accessed OAHS was similar to the overall percentage distributions of all clients in these subpopulations.

**Table 2:** Transgender women, cisgender women, and cisgender men served by the HRSA RWHAP who received OAHS, 2016.

Sociodemographic characteristics	Transgender women (*N* = 4,195; 64.2% of all transgender women)	Cisgender women (*N* = 98,550; 68.8% of all cisgender women)	Cisgender men (*N* = 253,724; 61.6% of all cisgender men)
**Age, median (IQR)**	38 (30–48)	48 (38–56)	47 (35–55)
**Race/Ethnicity**^**a**^**, *n* (%)**			
Black	2,200 (53.0)	61,015 (62.1)	107,067 (42.4)
Hispanic/Latino	1,289 (31.0)	19,940 (20.3)	63,704 (25.2)
White	464 (11.2)	15,010 (15.3)	74,300 (29.4)
Other	197 (4.8)	2,310 (2.3)	7,477 (3.0)
**Poverty level, *n* (%)**			
0%–100%	3,120 (75.6)	70,542 (72.9)	150,004 (60.6)
101%–138%	426 (10.3)	10,102 (10.5)	29,844 (12.1)
139%–250%	396 (9.6)	11,329 (11.7)	41,318 (16.7)
251%–400%	127 (3.1)	3,646 (3.8)	17,895 (7.2)
>400%	56 (1.4)	1,082 (1.1)	8,430 (3.4)
**Health care coverage, *n* (%)**			
Private employer	121 (2.9)	6,949 (7.1)	26,762 (10.6)
Private individual	218 (5.2)	5,277 (5.4)	21,294 (8.5)
Medicare	232 (5.6)	9,011 (9.2)	26,804 (10.6)
Medicaid	1,945 (46.7)	40,580 (41.4)	71,305 (28.3)
Medicare and Medicaid	232 (5.6)	7,981 (8.2)	17,015 (6.7)
Veterans Administration	2 (<0.1)	110 (0.1)	381 (0.2)
Indian Health Service	1 (<0.1)	42 (<0.1)	81 (<0.1)
Other plan	72 (1.7)	1,433 (1.5)	4,258 (1.7)
No coverage	1,001 (24.0)	17,089 (17.5)	58,835 (23.4)
Multiple coverages	340 (8.2)	9,484 (9.7)	25,160 (10.0)
**Housing status, *n* (%)**			
Stable	3,259 (78.9)	86,588 (89.1)	215,583 (86.6)
Temporary	529 (12.8)	7,000 (7.2)	22,144 (8.8)
Unstable	343 (8.3)	3,596 (3.7)	11,322 (4.6)
**Region, *n* (%)**			
Northeast	1,275 (31.0)	25,844 (27.6)	51,271 (21.1)
Midwest	543 (13.2)	10,411 (11.1)	33,816 (13.9)
South	1,474 (35.9)	48,618 (51.8)	113,031 (46.6)
West	817 (19.9)	8,929 (9.5)	44,609 (18.4)
**Number of OAHS visits, median (IQR)**	8 (4–17)	8 (4–16)	7 (3–13)

^a^Hispanics/Latinos can be of any race

Transgender women missing data on race/ethnicity (*n* = 45), poverty level (*n* = 70), health care coverage (*n* = 31), and housing status (*n* = 64)

Cisgender women missing data on race/ethnicity (*n* = 275), poverty level (*n* = 1,849), health care coverage (*n* = 594), and housing status (*n* = 1,366)

Cisgender men missing data on race/ethnicity (*n* = 1,176), poverty level (*n* = 6,233), health care coverage (*n* = 1,829), and housing status (*n* = 4,675)

**Abbreviations:** HRSA RWHAP, Health Resources and Services Administration’s Ryan White HIV/AIDS Program; IQR, interquartile range; OAHS, outpatient ambulatory health services

### Retention in care

Overall, a lower percentage of transgender women were retained in care (79.8%), compared with cisgender women (83.7%), and slightly lower than cisgender men (81.0%; *p* < 0.001; Table 3). Among Whites, the percentage of clients retained in care was similar for transgender women, cisgender women, and cisgender men (80.5% transgender women, 80.8% cisgender women, 80.3% cisgender men, *p* = 0.46). Comparatively, among Blacks, only 76.1% of transgender women were retained in care, compared with 83.1% of cisgender women and 79.5% of cisgender men (*p* < 0.001). Among Hispanics/Latinos, transgender women (85.1%) had lower retention than cisgender women (88.0%) and slightly higher retention than cisgender men (84.4%, *p* < 0.001).

**Table 3:** Retention in care and viral suppression among transgender women, cisgender women, and cisgender men served by the HRSA RWHAP, by race/ethnicity, 2016.

Race/ethnicity	Retention in care			Viral suppression		
	Yes	No	*p*-value	Yes	No	*p*-value
**All clients, *n* (%)**						
Transgender women	3,050 (79.8)	770 (20.2)	<0.001	3,124 (79.0)	829 (21.0)	<0.001
Cisgender women	77,035 (83.7)	15,052 (16.4)		79,903 (84.0)	15,178 (16.0)	
Cisgender men	188,612 (81.0)	44,349 (19.0)		207,749 (85.4)	35,641 (14.6)	
**White clients, *n* (%)**						
Transgender women	347 (80.5)	84 (19.5)	0.46	362 (82.3)	78 (17.7)	<0.001
Cisgender women	11,234 (80.8)	2,675 (19.2)		12,441 (86.3)	1,976 (13.7)	
Cisgender men	54,930 (80.3)	13,469 (19.7)		64,208 (90.1)	7,036 (9.9)	
**Black/African American clients, *n* (%)**						
Transgender women	1,504 (76.1)	472 (23.9)	<0.001	1,553 (75.1)	408 (32.2)	<0.001
Cisgender women	47,255 (83.1)	9,640 (16.9)		48,692 (82.6)	10,280 (17.4)	
Cisgender men	77,729 (79.5)	20,003 (20.5)		83,258 (80.7)	19,950 (19.3)	
**Hispanic/Latino clients, *n* (%)**						
Transgender women	1,028 (85.1)	180 (14.9)	<0.001	1,034 (85.0)	182 (15.0)	<0.001
Cisgender women	16,615 (88.0)	2,272 (12.0)		16,645 (86.5)	2,604 (13.5)	
Cisgender men	49,798 (84.4)	9,242 (15.7)		53,112 (87.4)	7,632 (12.6)	

Retention in care was based on data for PLWH who had at least 1 OAHS visit by September 1 of the measurement year, with a second visit at least 90 days after

Viral suppression was based on data for PLWH who had at least 1 OAHS visit during the measurement year and whose most recent viral load test result was <200 copies/mL.

**Abbreviations:** HRSA RWHAP, Health Resources and Services Administration’s Ryan White HIV/AIDS Program; OAHS, outpatient ambulatory health services; PLWH, people living with HIV

After adjustment for confounding, Black transgender women were less likely to be retained in care than Black cisgender women (adjusted prevalence ratio [aPR]: 0.95, 95% CI: 0.92–0.97, *p <* 0.001; Table 4). However, no statistically significant association was observed comparing Black transgender women to Black cisgender men (aPR: 0.98, 95% CI: 0.96–1.01, *p* = 0.21). Among Whites and Hispanic/Latino clients, the association between gender and viral suppression was not statistically significant after adjustment for confounding.

**Table 4:** Association between gender and retention in care and viral suppression in the HRSA RWHAP, by race/ethnicity, 2016.

Race/ethnicity	Retained in care				Virally suppressed			
	cPR (95% CI)	*p*-value	aPR (95% CI)	*p*-value	cPR (95% CI)	*p*-value	aPR (95% CI)	*p*-value
**White clients**								
Transgender women	1.00 (0.95–1.04)	0.89	1.03 (0.98–1.07)	0.29	1.17 (0.70–1.96)	0.54	0.84 (0.50–1.43)	0.53
Cisgender women	1.00		1.00		1.00		1.00	
Transgender women	1.00 (0.96–1.05)	0.92	1.04 (0.99–1.09)	0.08	0.91 (0.87–0.95)	<0.001	0.94 (0.57–1.55)	0.81
Cisgender men	1.00		1.00		1.00		1.00	
**Black/African American clients **								
Transgender women	0.92 (0.89–0.94)	<0.001	0.95 (0.92–0.97)	<0.001	0.88 (0.67–1.15)	0.34	0.55 (0.41–0.73)	<0.001
Cisgender women	1.00		1.00		1.00		1.00	
Transgender women	0.96 (0.93–0.98)	<0.001	0.98 (0.96–1.01)	0.21	0.93 (0.91–0.95)	<0.001	0.55 (0.42–0.73)	<0.001
Cisgender men	1.00		1.00		1.00		1.00	
**Hispanic/Latino clients**								
Transgender women	0.97 (0.94–0.99)	<0.01	0.99 (0.97–1.02)	0.46	1.47 (0.81–2.66)	0.20	1.15 (0.63–2.10)	0.65
Cisgender women	1.00		1.00		1.00		1.00	
Transgender women	1.01 (0.99–1.03)	0.47	1.02 (1.00–1.04)	0.12	0.97 (0.95–1.00)	0.02	0.82 (0.46–1.46)	0.50
Cisgender men	1.00		1.00		1.00		1.00	

Retention in care was based on data for PLWH who had at least 1 OAHS visit by September 1 of the measurement year, with a second visit at least 90 days after.

Viral suppression was based on data for PLWH who had at least 1 OAHS visit during the measurement year and whose most recent viral load test result was <200 copies/mL.

**Abbreviations:** HRSA RWHAP, Health Resources and Services Administration’s Ryan White HIV/AIDS Program; aPR, adjusted prevalence ratio; CI, confidence interval; cPR, crude prevalence ratio; OAHS, outpatient ambulatory health services; PLWH, people living with HIV

### Viral suppression

Overall, a lower percentage of transgender women (79.0%) were virally suppressed than both cisgender women (84.0%) and cisgender men (85.4%, *p* < 0.001; Table 3). This trend was consistent across all racial/ethnic groups.

Black transgender women were approximately 45% less likely to reach viral suppression than both Black cisgender women (aPR: 0.55, 95%I CI: 0.41–0.73, *p <* 0.001) and Black cisgender men (aPR: 0.55, 95% CI: 0.42–0.73, *p <* 0.001; Table 4). Among Whites and Hispanic/Latino clients, the association between gender and viral suppression was not statistically significant after adjustment for confounding.

## Discussion

The HRSA RWHAP serves over 6,500 transgender women, the majority of whom are Black and living at or below the FPL. In this analysis of HIV clinical outcomes among RWHAP clients, Black transgender women were significantly less likely to be retained in care than Black cisgender women and significantly less likely to reach viral suppression than both Black cisgender men and Black cisgender women.

In the absence of national surveillance data on HIV among transgender women, observational studies have been the primary source of information on transgender women living with HIV. Although observational studies have assessed overall patterns of HIV clinical outcomes among transgender women and racial disparities in HIV clinical outcomes among transgender women and cisgender PLWH, the relatively small sizes of these studies were not adequately powered to detect these disparities. With over 6,000 transgender women, the RWHAP data set used in this analysis is the largest available data source in the US that can assess patterns of HIV clinical outcomes among transgender women and the impact of racial/ethnic disparities on the association between gender and HIV clinical outcomes. Overall, the findings from this analysis are consistent with current evidence from smaller, observational studies [18–22], providing important corroboration of previously published findings.

Existing research indicates that Black transgender women are disproportionately impacted by the HIV epidemic; approximately half of transgender women diagnosed with HIV in the US from 2009 to 2014 were Black [36]. The results from this analysis suggest that this disproportionate burden extends beyond diagnoses to the HIV clinical outcomes of retention in care and viral suppression. Black transgender women living with HIV face the intersectional dilemmas of race, gender identity, and HIV infection, which may increase barriers to seeking and sustaining engagement in healthcare. In addition, Black transgender women also face the systems-level barriers of stigma and discrimination, lack of sufficient housing, insufficient transportation, and unemployment or underemployment [10,13,23,37,38]. These barriers can negatively impact HIV-related clinical indicators, including low rates of HIV testing, missed appointments, and nonadherence to ART, which ultimately can lead to not reaching viral suppression. In addition, these barriers can influence uptake of pre-exposure prophylaxis (a biomedical intervention to prevent HIV acquisition) for transgender women [12,39–41]. The pronounced disparities in HIV clinical outcomes observed in this analysis between Black transgender women and Black cisgender PLWH may be attributable to these barriers.

In this study, transgender women were, on average, 10 years younger than cisgender women and cisgender men. Nearly 40% of transgender women were under 35 years of age, compared with 16% of cisgender women and 23% of cisgender men. Age has been strongly correlated with HIV clinical outcomes, with younger PLWH less likely to reach viral suppression than older PLWH [25,32]. Young PLWH experience unique barriers to accessing HIV care, including violence, rejection, discrimination, and confidentiality related to HIV status and sexual orientation [42,43]. Additional factors influencing care decisions for young PLWH are the structural challenges faced when accessing and navigating the healthcare landscape, such as cost for care or co-pays, lack of transportation, low health literacy, competing social and economic priorities, and limited educational attainment [42]. The driving force behind this difference in the age distribution between transgender women, cisgender women, and cisgender men in the RWHAP deserves further study. Potential explanations include bias because of undercounting of older transgender people or differential mortality rates by gender [44]. Young transgender women may especially experience the complex combination of challenges experienced by young PLWH and those of older transgender PLWH in accessing HIV care and treatment, including stigma, unstable housing, and poverty [10,11,17,20].

Although the RWHAP provides care and support services to over 500,000 low-income PLWH, reaching over half of people living with diagnosed HIV in the US, it is not representative of all PLWH, nor all transgender women [25]. Compared with all people with diagnosed HIV in the US, RWHAP clients in care have higher rates of viral suppression; transgender women in the RWHAP also have higher rates of viral suppression than has been observed in other, smaller studies [18–21,25,32]. These differences can likely be attributed to the strengths of the RWHAP’s comprehensive system of care. However, within the RWHAP, this study identified disparities in viral suppression for Black transgender women. These disparities may be more pronounced in environments with lower overall levels of viral suppression.

Historically, HIV prevention and treatment programs aggregated transgender women and cisgender men who have sex with men based on assumptions that these groups had similar risk factors and could be reached through similar interventions [45,46]. More recently, however, HIV prevention and treatment efforts have increasingly recognized the need for the development of transgender-specific outreach and intervention activities [40,47]. Although comparisons of transgender PLWH to cisgender PLWH are important to measure progress toward reducing disparities, within group comparisons are essential to assess whether targeted interventions are actually improving health outcomes for transgender women. The RWHAP’s collection of nuanced, multi-category gender data and the multi-category gender analysis methods presented within this paper can serve as a powerful tools in measuring disparities and intervention development. Future studies should extend the work presented within this paper to transgender men and gender diverse people with HIV. Finally, it is essential to ensure people who identify as transgender are reported as such in the analytic data source [24].

Despite the strengths of this study, such as the large data set and the ability to conduct a multi-category gender analysis, there are limitations. First, RWHAP data are collected for administrative, not research, purposes. As a result, the data set only includes a limited set of data elements and not all clinical outcome measures are available for all RWHAP clients. The limited nature of the data set precludes the inclusion of all potential confounders in analyses and limits some time-varying covariates to data collection at a single point in time. Second, clinical outcome measures, including retention and viral suppression, are only reported to the RWHAP for the approximately two-thirds of RWHAP clients engaged in RWHAP-supported outpatient medical care. For the 40% of RWHAP clients who only accessed RWHAP-funded services that support engagement in HIV medical care, the status of their engagement in HIV medical care is unknown—they may be engaged in HIV primary care in non–RWHAP-funded settings, may sporadically access HIV care, or may be “lost to care.” Beyond the RWHAP, transgender women are less likely to engage in outpatient HIV medical care than cisgender men and women [12,20,32]. If the same patterns of care engagement hold within the RWHAP, then the results of this analysis could underestimate the gender disparities experienced by transgender women in the broader RWHAP. Third, RWHAP client-level data do not permit the assessment of time-to-event outcomes, such as time to dropping out of care or time to virologic failure or assessment of the quality of HIV care, at the level of granularity necessary for this analysis focused on transgender women. Therefore, it is important that RWHAP grant recipients and other HIV care providers monitor these outcomes within their client populations.

Recognizing the high burden of HIV among transgender women, especially Black transgender women, the National HIV/AIDS Strategy for the United States: Updated to 2020 (NHAS 2020) identified transgender women as a key priority population and includes a developmental indicator to increase the percentage of transgender women in HIV medical care who are virally suppressed to at least 90% [48,49]. The disparities identified in this study indicate that more work is needed to reach the NHAS 2020 goals including additional research to further explore and understand the mechanisms by which transgender women experience poor clinical outcomes and identify factors that may contribute to positive outcomes, such as peer navigation or other support services. HRSA HAB currently supports evaluation studies to address these questions, as well as facilitate more advanced analysis of RWHAP data through collection of more diverse data elements via a sampled chart abstraction. Developing and testing interventions for Black transgender women may significantly improve viral suppression among transgender women, and HRSA HAB supports the implementation of evidence-informed interventions specifically designed to improve health outcomes for transgender women living with HIV [50]. Additionally, HRSA HAB’s Special Projects of National Significance has supported the development and evaluation of new interventions to improve timely entry to, engagement in, and retention in quality HIV care for transgender women of color living with HIV [51]. The RWHAP is committed to providing opportunities for transgender women to obtain leadership skills through the Building Leaders of Color (BLOC) initiative. The purpose of BLOC is to provide training to people of color living with HIV so they can participate in RWHAP planning bodies, medical and support care teams, boards of directors, and other mobilization efforts [52]. BLOC includes training dedicated for transgender people of color. All of the RWHAP initiatives, in combination with the high quality care and treatment by RWHAP providers, have contributed to the evident increase in viral suppression among transgender women, from 65% in 2012 to 79% in 2016, and will be an important component to the continued reduction in disparities among transgender women living with HIV in the US [30,31].

This analysis provides insight into the HIV clinical outcomes and disparities among the underserved community of transgender women living with HIV. Within the RWHAP, disparities persist in HIV clinical outcomes between transgender women and cisgender PLWH, especially for Black transgender women. HRSA HAB is uniquely positioned to monitor HIV clinical outcomes, including retention in care and viral suppression, among RWHAP clients, with the data to identify disparities and the resources to develop and support initiatives to mitigate those disparities. By continually measuring progress toward eliminating these disparities while also developing, testing, and implementing evidence-informed interventions, the RWHAP has the opportunity to improve the health of transgender women living with HIV. Closing these gaps in HIV medical care for transgender women and ensuring timely entry, engagement, and retention in quality care for all PLWH are critical to ending the HIV epidemic.

## Supporting information

S1 STROBE Checklist STROBE statement. Checklist of items that should be included in reports of cross-sectional studies. STROBE, Strengthening the Reporting of Observational Studies in Epidemiology.
(DOCX)

## Abbreviations

PR: adjusted prevalence ratio
ART: antiretroviral treatment
BLOC: Building Leaders of Color
CI: confidence interval
FPL: federal poverty level
FTM: female-to-male
HRSA HAB: Health Resources and Services Administration’s HIV/AIDS Bureau
IQR: interquartile range
IRB: institutional review board
MTF: male-to-female
NHAS 2020: National HIV/AIDS Strategy for the United States: Updated to 2020
OAHS: outpatient ambulatory health services
OMB: Office of Management and Budget
PLWH: people living with HIV
RSR: RWHAP Services Report
RWHAP: Ryan White HIV/AIDS Program
STROBE: Strengthening the Reporting of Observational Studies in Epidemiology
